# Shared Medical Appointments and the Quintuple Aim of Health Care: A State of the Art Literature Review

**DOI:** 10.1177/15598276261433486

**Published:** 2026-03-11

**Authors:** John Stevens, Willow Firth

**Affiliations:** 1 The Australian Society of Lifestyle Medicine (ASLM), Lifestyle, 4571 Medicine Southern Cross University, Lismore, NSW, Australia (JS); 2 Credentialled Diabetes Educator & Exercise Physiologist & Indigenous Trauma Informed Clinician, Waminda- South Coast Aboriginal Womens Health and Wellbeing Organisation (WF), Nowra, NSW, Australia

**Keywords:** quintuple aim of health care, shared medical appointments, group consultations, literature review

## Abstract

The escalating global burden of chronic disease, increasing health inequities, and provider burnout require innovative care models. Shared medical appointments (SMAs) are group-based interventions with potential to advance the Quintuple Aim framework: patient experience, population health, cost reduction, provider wellbeing, and health equity. This review critically synthesizes evidence regarding SMAs’ capacity to meet the Quintuple Aim in lifestyle and chronic disease care. Peer-reviewed studies, including systematic reviews, RCTs, and observational research, were selected through biomedical database searches including Medline CINHAL Joanna Briggs Institute and PubMed. Criteria: SMAs (and other related terms) addressing one or more Quintuple Aim domains. Strong evidence supports enhanced patient satisfaction, timely access, and communication in SMAs. Population health shows improved outcomes for diabetes, cardiovascular disease, COPD, and prenatal care. Cost-effectiveness is achievable with effective design. Provider wellbeing benefits include reduced burnout and improved teamwork. SMAs advance health equity, providing effective models for Indigenous, rural, and disadvantaged cohorts. SMAs align robustly with the Quintuple Aim and demonstrate multi-domain impacts. Chronic disease and lifestyle medicine practitioners and health services should consider their broad adoption and ongoing local adaptation.

## Introduction

The health care landscape faces unprecedented challenges, including rising costs, increasing prevalence of chronic diseases, provider burnout, and persistent health inequities. To address these complex issues, the Institute for Health care Improvement developed the Triple Aim framework in 2008, focusing on enhancing patient experience, improving population health, and reducing per capita costs.^1^ This framework subsequently evolved into the Quadruple Aim with the addition of improving health care provider wellbeing,^2^ and most recently expanded to the Quintuple Aim, which explicitly incorporates health equity as a central pillar.^3,4^ The Quintuple Aim now provides a comprehensive framework encompassing: (1) enhancing patient experience, (2) improving population health, (3) reducing health care costs, (4) improving provider wellbeing, and (5) advancing health equity.^3^

Shared medical appointments (SMAs), also known in the literature as group visits, group medical appointments or group medical visits (for clarity the term Shared Medical Appointment, SMA, will be used throughout this review unless the intext citations refers to one of the other terms) represent an innovative health care consultation process and model of care with potential to simultaneously address all five quintuple aims. SMAs are comprehensive medical visits conducted with groups of patients who typically share similar health conditions or health care needs.^5^ During these appointments, patients receive individual clinical consultations within a supportive group learning environment, usually lasting 90-120 minutes with 6-20 participants.^1,6^

## Method

This state-of-the-art literature review examined peer-reviewed evidence demonstrating how SMAs meet each of the quintuple aims of health care, synthesizing findings from systematic reviews, randomized controlled trials, and other rigorous research studies to evaluate the effectiveness of this care delivery model.

Peer-reviewed studies, with no publication date limitations, including systematic reviews, RCTs, and observational research, were selected through biomedical database searches that included Medline, CINHAL, Joanna Briggs Institute and PubMed. Search Criteria were: SMAs (and other related terms) addressing one or more Quintuple Aim domains.

The synthesis of the outcomes from this search is presented below organized by the Quintuple Aim framework.

## Patient Experience of Care (Aim 1)

### Patient Satisfaction and Quality Perceptions

Extensive evidence demonstrates that SMAs are associated with high levels of patient satisfaction and improved perceptions of care quality. A systematic review by Wadsworth et al (2019) examining SMAs in primary care settings found that patients participating in SMAs reported significantly greater overall satisfaction with their care compared to those receiving traditional individual appointments.^1^ Multiple studies within this review demonstrated that SMA patients were more likely to rate their overall quality of care as excellent and to feel that their care was meeting all their needs.^1,7^

Heyworth et al (2014) conducted a large cross-sectional study using mailed questionnaires measuring patient satisfaction among 40% of SMA patients and 31% of usual care patients across a multispecialty practice.^7^ In adjusted analyses, SMA patients were significantly more likely to rate their overall satisfaction with care as “very good” compared to usual care counterparts).^7^ The study found that SMA patients consistently rated their care as more accessible and more sensitive to their needs.^7^

Across all 26 articles in the Wadsworth systematic review that reported patient satisfaction outcomes, no studies showed significant decreases in patient perceptions of quality of care in SMAs.^1^ Patients particularly valued the extended time available for discussion with clinicians, the supportive group environment, and the opportunity to learn from peers’ questions and experiences.^1,6^

### Enhanced Access to Care

SMAs consistently demonstrate improved access to care, addressing a critical challenge in many health care systems. Heyworth et al (2014) found that SMA patients reported significantly more timely access to care, reinforcing anecdotal experiences described by practices adopting SMAs nationally.^7^ Primary care departments offering SMAs as an alternative to routine care enabled patients to be seen more promptly than would otherwise be possible.^7^

A systematic review by Tang et al (2024) examining SMAs delivered in primary care for long-term conditions confirmed that SMAs may improve access to care and deliver care that patients find sensitive to their needs.^8^ The review noted that SMAs provide a potential solution for practices experiencing difficulty accommodating patients in a timely fashion, particularly important given the growing numbers of patients requiring chronic disease management.^8^

### Patient–Clinician Communication and Relationship Quality

The patient–clinician dynamic that emerges during SMAs has been consistently positive across studies. SMAs demonstrated quantitative advantages over individual visits in domains ranging from improved communication to overall satisfaction.^1,9^ In SMA environments, more time was allocated to discuss health care issues with clinicians compared to traditional individual visits, and physicians were perceived as less hurried.^1,9^ One study indicated that SMA experiences resulted in markedly enhanced trust in patients’ primary care physicians.^10^

However, some studies have identified nuances in communication satisfaction. Heyworth et al (2014) found that while overall satisfaction was higher in SMAs, usual care patients rated higher levels of satisfaction with their relationship with their clinician.^7^ This finding suggests the importance of enhancing patient–clinician communication strategies within SMAs to maximize this aspect of patient experience.^7^

## Population Health Outcomes (Aim 2)

### Diabetes and Weight Management

Diabetes represents an extensively studied condition in SMA research, with substantial evidence demonstrating clinical benefits. A meta-analysis by Edelman et al (2014) examined SMAs for patients with diabetes and found that hemoglobin A1c improved by approximately 0.6 percentage points, and systolic blood pressure by about 5 mmHg, with both findings being statistically significant.^11^ LDL cholesterol improved by approximately 7 mg/dL, though this was not statistically significant.^11^

Tang et al (2024) conducted a systematic review and meta-analysis of 29 unique randomized controlled trials, with 16 trials recruiting patients with diabetes.^8^ The meta-analysis showed that participants in SMA groups had significantly lower diastolic blood pressure than those in usual care (d = −0.086, 95% CI = −0.16 to −0.02, n = 10) (*P* = 0.014).^8^ While the overall meta-analysis for HbA1c did not show statistically significant differences (d = −0.091, 95% CI = −0.27 to 0.09), substantial heterogeneity was observed across studies.^8^

Individual studies have demonstrated more pronounced effects. A collaborative diabetes SMA study in an urban setting showed statistically significant improvements in HbA1c (*P* < .001) and systolic blood pressure (*P* = .004), with more patients achieving LDL-C goals of ≤100 mg/dL (*P* < .001) and receiving appropriate preventive care including aspirin therapy and pneumonia vaccination (*P* < .001).^12^ The study also found significant improvements in diabetes-related emotional distress and diabetes knowledge scores.^12^

Kirsh et al (2007) demonstrated the feasibility of implementing effective SMAs using an interdisciplinary team approach for high-risk diabetes patients.^13^ Patients achieved benefits in terms of cardiovascular risk reduction, with the SMA group demonstrating greater improvements in glycemic control and blood pressure management compared to usual care.^13^

### Cardiovascular Disease and Hypertension

SMAs have shown promising results for cardiovascular disease management and hypertension control. Kirk et al (2017) evaluated a pharmacist-led SMA for veterans with hypertension and found that 76.2% of participants had a reduction in systolic blood pressure with an overall average decrease of −8.3 mmHg (*P* = .02).^14^ The proportion of veterans with controlled blood pressure (<140/90 mmHg) increased from 14.3% at baseline to 42.9% during the SMA period (*P* = .03).^14^

A systematic review by Cunningham et al (2021) found that group care patients were more likely to have had referrals for American Diabetes Association process-of-care indicators and recommended preventive procedures such as foot and retinal eye examinations.^15^ Patients randomized to group care also demonstrated higher rates of influenza and pneumonia vaccinations.^15^

### Prenatal Care Outcomes

CenteringPregnancy®, a specialized SMA model for prenatal care, has accumulated substantial evidence of improved maternal and neonatal outcomes. A quasi-experimental study examining CenteringPregnancy outcomes found that the model significantly improved women’s access to prenatal care and reduced rates of preterm birth and perinatal death compared to traditional prenatal care.^16^ The study demonstrated that CenteringPregnancy resulted in longer gestation periods and better infant birth weight outcomes.^16^

CenteringPregnancy program reported a 4% rate of preterm birth compared to the national average of approximately 10%, and 92% breastfeeding at discharge compared to the national average of 83%.^17^ The program demonstrated a track record of improved outcomes, reduced health care costs, and higher patient satisfaction levels.^17^

A systematic review by Cunningham et al (2021) identified nine studies focused on pregnancy outcomes in group prenatal care models.^15^ Compared with individual care, group visits demonstrated potential to improve patient experience, health outcomes, and costs, with no adverse effects associated with group health care delivery in randomized controlled trials.^15^

### Chronic Obstructive Pulmonary Disease

SMAs have demonstrated effectiveness for patients with chronic obstructive pulmonary disease (COPD). Zhang et al (2022) conducted a prospective randomized study of 116 patients with COPD post-discharge, randomly assigning them to either a control group or group visit intervention.^18^ Results showed that healthy lifestyle and self-efficacy scores were significantly higher in the group visit intervention after the intervention compared to before and compared to the control group.^18^ Lung function improved in both groups, with greater improvement in the group visit group.^18^ Importantly, the frequency of outpatient emergency services and hospitalizations due to acute COPD exacerbations was lower in the group visit group compared with the control group.^18^

### Mental Health Conditions

Emerging evidence suggests SMAs may be beneficial for mental health conditions. A study on group medical visits for anxiety and depression in primary care found significant and meaningful reductions in Patient Health Questionnaire-9 (PHQ-9) and Generalized Anxiety Disorder-7 (GAD-7) scores from baseline to last visit attended, with decreases of 5.13 and 5.26 points respectively among those who attended at least one visit.^19^ A qualitative study examining acceptability and feasibility of SMAs for anxiety and depression found that main benefits reported were peer support, support for self-management, and quicker access to care.^20^

### Preventive Care and Health Maintenance

SMAs facilitate delivery of comprehensive preventive care services. Cunningham et al (2021) reported that group visit patients were more likely to engage in cancer screenings and had higher rates of influenza and pneumonia vaccinations compared to individual care patients.^15^ The extended time available in SMAs allows for more thorough attention to routine health maintenance and performance measures, including medication reviews, preventive health procedures, and patient education.^1^

## Cost Reduction and Health care Utilization (Aim 3)

### Economic Evaluations

Evidence regarding the cost-effectiveness of SMAs presents a mixed but generally favorable picture. Seesing et al (2015) conducted a randomized controlled trial examining cost-effectiveness of SMAs for neuromuscular patients, finding no substantial differences between SMAs and individual visits in terms of costs per quality-adjusted life-year (QALY) gained (incremental cost-effectiveness.^21^ However, sensitivity analyses demonstrated that SMAs became cost-effective with a minimum of 6 patients per session and when 75% of patients saw their treating physician.^21^ This finding indicates that SMAs can be a means to increase physician productivity without compromising quality of care under specific implementation conditions.^21^

A Duke University study examining SMAs for diabetes found that for patients who attended the majority of SMA sessions, the model appeared more cost-effective than usual care.^22^ The analysis included costs of hospital admissions, emergency room visits, primary care visits, specialist visits, laboratory tests, medications, and travel costs.^22^ Primary care costs statistically significantly decreased on average by $297-326 during the SMA period, likely because SMAs replaced patients’ non-regular visits with their primary care physicians.^22^

Australian economic modeling suggests substantial potential cost savings. One analysis indicated that achieving similar weight loss outcomes through SMAs compared to individual consultations reduced the number of total visits to the GP and in 2019 could cost approximately $300 per patient for 6 sessions with 10 patients per group, compared to $670 for 18 individual consultations needed for the equivalent weight loss outcomes as the SMA patients while simultaneously saving 37 hours of GP time for the equivalent group of patients.^23,24^

### Health Care Utilization Patterns

Multiple studies demonstrate that SMAs are associated with reduced health care utilization in high-cost settings. A review of SMA studies for chronic conditions found that all studies showed fewer hospital admissions in the SMA group, though the difference was not always statistically significant.^25^ The efficiency gains from SMAs are considerable, with productivity increases typically 300% or higher in drop-in SMA models.^26^

For COPD specifically, Zhang et al (2022) found that group visits reduced the frequency of outpatient emergency services and hospitalizations due to acute COPD exacerbations, leading to lower medical costs during the post-discharge transition period.^18^ Similarly, prenatal group care models have demonstrated lower rates of preterm births and neonatal intensive care unit admissions, representing significant cost savings associated with improved maternal and child health outcomes.^27^

### Provider Efficiency and Productivity

SMAs enhance provider efficiency through multiple mechanisms. The model allows physicians to typically see 8-10 patients in a 90-120 minute session compared to individual appointments, representing a 300% productivity improvement for routine follow-up care.^26^ This efficiency is achieved without compromising quality, as each patient receives individualized attention during the group session.^26^

Economic benefits extend beyond direct productivity gains. SMAs reduce repetition of information and advice, as education provided to one patient benefits all group participants.^24,28^ The addition of supporting contributions from other health professionals allows general practitioners to focus on specific medical issues while team members address other aspects of care.^28^ Together with improved patient outcomes and potentially reduced requirements for ongoing visits, this leads to increased cost-effectiveness.^28^

## Provider Wellbeing and Experience (Aim 4)

### Reduction of Burnout

Physician and health care provider burnout represents a critical challenge in modern health care systems. Thompson-Lastad and Gardiner (2020) argue that SMA visits should be considered among workplace-based interventions for reducing burnout, given their potential benefits to clinician wellbeing.^29,30^ The authors suggest that SMA programs with adequate institutional support may be beneficial for preventing burnout and improving retention among clinicians and health care teams more broadly.^30^

A qualitative study examining SMAs for anxiety and depression found that participants anticipated SMAs could benefit health care providers who are often “busy” and “extremely overworked” by reducing clinician burnout and improving the quality of care provided to each patient.^20^ This perception aligns with evidence that workplace-based interventions focusing on improved teamwork and organizational support are associated with reduced burnout.^31,32^

### Enhanced Job Satisfaction

Multiple studies report that clinicians offering SMAs experience increased job satisfaction. Thompson-Lastad and Gardiner (2020) note that nearly all SMA clinicians who continue offering individual care report that replacing some individual visits with SMA increased their job satisfaction, in part because it provided more variety in their work schedules and time to develop more trusting relationships with patients.^30^ The extended time available in SMA makes health care interactions more enjoyable for staff and patients alike, with activities such as cooking together and learning mind-body practices often included.^30^

Providers report that SMAs allow them to practice the kind of caring, holistic medicine that inspired them to become doctors, with substantial savings in time, energy, and money.^33^ The model offers opportunities for better management of waiting lists, reduced repetition, and a chance to get to know patients better in an interactive setting, leading to improved provider efficiency and work satisfaction.^24,28^

### Team-Based Care Benefits

SMAs provide opportunities for enhanced team-based care through interdisciplinary collaboration.^30^ SMAs often include continuity of staffing, with primary care providers typically supported by one or more team members such as health educators, behavioral health clinicians, or community health workers.^30^ Some models include multiple practitioners to facilitate provision of whole-person care, such as a psychologist and nurse-practitioner, or an acupuncturist and physician.^30^

Research demonstrates strong relationships between teamwork climate and reduced burnout. One study found that each one-point increase in teamwork climate was associated with 16% lower odds of high emotional exhaustion, 26% lower odds of high depersonalization, and 20% lower odds of burnout.^32^ Funk et al (2024) found that clinicians who reported having someone on their care team routinely schedule follow-up appointments for patients with complex chronic illnesses reported significantly lower stress and burnout.^34^

SMAs facilitate this collaborative approach by bringing together different health care professionals in a coordinated setting. The interdisciplinary perspectives draw on all staff members’ strengths, providing opportunities for team members to co-facilitate and deliver comprehensive care.^30^ This teamwork approach reduces feelings of isolation commonly experienced by individual practitioners and enhances the overall quality of care delivery.^29,30^

### Understanding Social Context and Addressing Social Determinants

SMAs enable clinicians to better understand and address patients’ social context and social determinants of health.^30^ The extended time and presence of peers facilitate patients sharing knowledge and experiences, reducing loneliness while providing clinicians, other health care staff, and peers the opportunity to provide referrals and follow-up with needed resources.^30^ These resources extend beyond health care services to include legal aid and public benefits programs addressing patients’ social needs.^30^

Perceiving that their workplace is equipped to address patients’ social needs has been correlated with lower physician burnout.^30,35^ Understanding and addressing patients’ social context may increase mutual trust between patients and clinicians, further enhancing provider satisfaction and reducing burnout.^30^

## Health Equity (Aim 5)

### Improving Access for First Nations Peoples

SMAs demonstrate promise for improving health care access and cultural appropriateness for First Nations peoples. Stevens et al (2016) examined SMAs for Aboriginal and Torres Strait Islander men in Northern New South Wales, finding unanimous positive satisfaction among participants.^36^ Patients particularly enjoyed the “yarn up” nature of SMAs with peer support, which reduced the “scary” and culturally “unnatural” nature of one-on-one consultations with general practitioners.^36^

The study found that sharing circles in a gender-specific environment align with common cultural practices in Aboriginal and Torres Strait Islander societies.^36^ The SMA concept capitalizes on the “sharing circle” concept and appears more natural than one-to-one medical consultations, which many described as intimidating.^36^ Aboriginal health workers trained to facilitate Shared Medical Appointments (SMAs) viewed this approach not as an innovation, but as a renewal of cultural practices and a way of enacting First Nations ways of Knowing, Being, and Doing. This model simultaneously supports cultural competence among Western-trained health professionals while improving accessibility and engagement in Aboriginal health services.^36,37^

One study from the Waminda—South Coast Aboriginal Women’s Health & Wellbeing Organisation, in NSW Australia showed that SMAs provided a process to embed their decolonizing model of care and to deliver a successful co-designed Type 2 Diabetes management and reversal program called DRAW (Diabetes Reversal in Aboriginal Women). The DRAW program resulted in HbA1c percentage reduction, on average, of .71% (*P* = .01), weight 3.83 kgs (*P* < .001) and Systolic Blood Pressure by 8.76 mm/hg (*P* < .001). The study reported that 28% of completing participants (n = 25) entered remission and remained in remission at the 12-month measure, while 72% of others obtained significant reductions in HbA1c that were also maintained at the 12-month measure.^38^

### Expanding Care in Rural and Underserved Communities

SMAs offer solutions for clinicians serving under-resourced and rural populations, where delivering effective chronic disease care in traditional 15-minute visits can feel nearly impossible.^39^ The model combines clinical care, education, and peer support in a group setting, addressing multiple barriers to care access simultaneously.^39^

A case study from the Snowy Valleys region in Australia demonstrated the viability of SMAs in rural and remote settings.^40^ Three trials focused on different populations: First Nations people with type 2 diabetes, patients with COPD, and women over 65 with osteoporosis.^40^ The COPD trial achieved an average of 8 participants per session and proved financially viable, while also providing substantial patient benefits including more time with the GP and health team, increased health literacy, and holistic multidisciplinary care.^40^

Benefits for rural communities include reduced GP wait times by servicing more patients simultaneously, addressing chronic disease in primary care settings before conditions escalate, facilitating multidisciplinary care in rural settings, and potentially reducing pressure on emergency departments and hospitals over time.^40^

### Telehealth and Virtual SMAs

The expansion of telehealth capabilities has created new opportunities for SMAs to enhance health equity by reaching geographically isolated populations. Patel et al (2020) evaluated patient–provider telemedicine encounters during group visits for diabetes and found they were feasible and acceptable as evidenced by systematic evaluation.^41^ The Telehealth Usability Questionnaire revealed that participants found telemedicine useful and easy to use (4.9/5.0, 4.4/5.0, respectively) with excellent interface, interaction, reliability, and satisfaction ratings.^41^ Importantly, there were no significant differences in clinical outcomes between in-person and telemedicine arms for HbA1c, blood pressure, weight, BMI, or attendance.^41^

Virtual group visits enable participation of up to 50 patients, substantially more than traditional in-person groups limited to 10-15 participants.^42^ This scalability combined with the convenience of remote participation can dramatically increase access to specialized care and lifestyle medicine programming for patients in remote areas or those with mobility challenges.^42^

Gizaw et al (2022) identified telemedicine as a key strategy to improve access to primary health care services in rural communities, noting that the provision of subspecialty services using telemedicine to remote and medically underserved populations provides improved access to subspecialty care.^43^ Virtual SMAs leverage this technology while maintaining the peer support and group learning benefits of the model.^41,42^

### Addressing Socioeconomic Barriers

SMAs may advance health equity by increasing access to guideline-concordant care for underserved communities.^5^ The model addresses multiple socioeconomic barriers simultaneously: extended appointment times provide comprehensive care that might otherwise require multiple visits, peer support reduces social isolation common in disadvantaged communities, group education enhances health literacy across diverse populations, and the efficiency of group appointments can make specialized care more accessible.^5,44^

Integrative health SMAs that incorporate complementary and integrative health care education and services may particularly benefit patient populations historically negatively impacted by health and health care disparities, as they increase patient access to services such as acupuncture, mindfulness, and yoga that are often inaccessible due to limited insurance coverage and high out-of-pocket costs.^44^

## Discussion

This literature review demonstrates substantial peer-reviewed evidence that shared medical appointments can effectively address all five aims of the Quintuple Aim framework. The evidence is particularly robust for patient experience and population health outcomes, with consistent findings of high patient satisfaction, improved access to care, and clinically meaningful improvements in chronic disease management across multiple conditions including diabetes, cardiovascular disease, prenatal care, COPD, and mental health conditions.

The evidence for cost reduction and health care utilization is promising though somewhat mixed, with studies demonstrating that cost-effectiveness depends significantly on implementation factors such as group size, attendance rates, and care model design. When implemented optimally, SMAs demonstrate clear efficiency gains and reduced utilization of high-cost services such as emergency departments and hospital admissions.

Provider wellbeing represents an emerging but encouraging area of SMA research. While direct studies of SMA impacts on burnout are limited, the theoretical framework and preliminary evidence suggest that the extended patient contact time, enhanced team-based care opportunities, and ability to address social determinants of health contribute to improved provider satisfaction and reduced burnout. This represents an important area for future research, particularly given the current crisis of health care workforce wellbeing.

The evidence for health equity is compelling, particularly regarding cultural appropriateness for First Nations peoples and improved access for rural and underserved communities. The integration of telehealth capabilities with the SMA model creates powerful opportunities to extend equitable access to high-quality care across geographic and socioeconomic barriers.

### Limitations and Research Gaps

Several limitations warrant acknowledgment. Substantial heterogeneity exists across SMA models, making direct comparisons challenging. Many studies lack long-term follow-up data, limiting understanding of sustained benefits. Economic analyses often focus on limited cost categories and may not capture full societal costs and benefits. Research on provider wellbeing impacts remains limited compared to patient-focused outcomes. Additionally, most studies are conducted in high-income countries, limiting generalizability to other settings.

Future research should focus on standardizing SMA implementation and measurement approaches, conducting rigorous economic evaluations from societal perspectives, investigating long-term sustainability and outcomes, examining impacts on provider wellbeing and workforce retention, and expanding evidence from diverse geographic and cultural settings. Implementation science research is particularly needed to understand barriers and facilitators to scaling SMAs across different health care systems.

### Implications for Practice and Policy

The evidence supports wider adoption of SMAs as a strategy to simultaneously advance multiple health care improvement goals. Health care organizations should consider SMAs as part of comprehensive approaches to chronic disease management, preventive care delivery, and health equity initiatives. Policy makers should ensure appropriate reimbursement mechanisms that recognize the value and efficiency of group-based care models.

Successful implementation requires adequate institutional support, including training for facilitators and clinicians, appropriate space and scheduling systems, clear billing and reimbursement processes, and strategies to ensure cultural appropriateness and accessibility for diverse populations. Organizations should develop quality metrics that capture the full range of SMA benefits across the quintuple aims, moving beyond traditional individual-visit metrics.

## Conclusion

Shared medical appointments represent a promising health care delivery innovation with substantial peer-reviewed evidence demonstrating effectiveness across all five aims of the Quintuple Aim framework. SMAs enhance patient experience through improved satisfaction, access, and quality of care. They improve population health outcomes across diverse chronic conditions and preventive care domains. When implemented optimally, SMAs demonstrate cost-effectiveness and improved health care utilization patterns. Evidence suggests benefits for provider wellbeing through enhanced job satisfaction, team-based care, and reduced isolation. Finally, SMAs advance health equity by providing culturally appropriate care for First Nations populations, expanding access in rural and underserved communities, and leveraging telehealth to overcome geographic barriers.

As health care systems worldwide grapple with rising demands, limited resources, provider burnout, and persistent inequities, SMAs offer a practical, evidence-based approach to simultaneously address these interconnected challenges. The model aligns well with contemporary emphases on patient-centered care, team-based delivery, population health management, and value-based reimbursement. With appropriate implementation support and continued research to refine best practices, SMAs have potential to contribute meaningfully to transforming health care delivery to meet the aspirations of the Quintuple Aim.

## Data Availability

As a literature review there is no data to be made available.*
